# Carta of Florence Against Ageism; No Place for Ageism in Healthcare

**DOI:** 10.1093/geroni/igad133

**Published:** 2024-02-29

**Authors:** Andrea Ungar, Antonio Cherubini, Laura Fratiglioni, Vânia de la Fuente-Núñez, Linda P Fried, Marlane Sally Krasovitsky, Mary Tinetti, Alana Officer, Bruno Vellas, Luigi Ferrucci

**Affiliations:** Division of Geriatrics and Intensive Care Medicine, Careggi Hospital and University of Florence, Florence, Italy; Geriatria, Accettazione geriatrica e Centro di ricerca per l’invecchiamento, IRCCS INRCA, Ancona, Italy; Aging Research Center, Department of NVS, Karolinska Institutet and Stockholm University, Stockholm, Sweden; Department of Neurobiology, Care Science and Society, Gerontology Research Center, Stockholm, Sweden; Independent Researcher, Barcelona, Spain; Butler Columbia Aging Center, Columbia University Mailman School of Public Health and Vagelos College of Physicians and Surgeons, New York City, New York, USA; Global Campaign to Combat Ageism, World Health Organization, Geneva, Switzerland; Department of Medicine, Yale School of Medicine, New Haven, Connecticut, USA; Demographic Change and Healthy Ageing, World Health Organization, Geneva, Switzerland; IHU HealthAge, University of Toulouse, INSERM CERPOP, Toulouse, France; Intramural Research Program of the National Institute on Aging, NIH, Baltimore, Maryland, USA

## Background and Scope

The United Nations (UN) Decade of Healthy Ageing (2021–2030) has identified ageism as a global obstacle that curtails older persons’ opportunities to contribute to society, realize their full potential, and lead a fulfilling life. The World Health Organization (WHO) established the Global Campaign to Combat Ageism (http://bit.ly/combatageism) to build a world for all ages by changing the way we think, feel, and act about age and aging. To provide evidence-based support for the Global Campaign to Combat Ageism, the WHO, in collaboration with other UN agencies, released the landmark “Global Report on Ageism” in 2021 (http://bit.ly/ageismreport). Recently, the U.S. National Academy of Medicine’s Global Roadmap for Healthy Longevity reinforced the need to address ageism and identified training, education, and new social infrastructure that values and enables the contribution of older adults as critical steps to promoting healthy longevity as one of the core missions of healthcare systems and society as a whole.

In spite of the massive growth in the number and percentage of older persons in the population and the rising prevalence of those affected by multimorbidity and disability, the care of older patients remains unsatisfactory and the medical practice relies mostly on a stand-alone (single) disease approach. As demonstrated in robust literature, ageism is widespread and has damaging effects. Ageism is in our institutions, our relationships, and ourselves. Pervasive ageism in healthcare negatively affects healthy survival and trajectories of health and well-being of older persons and curtails individuals’ capacity to contribute to societal goals. Thus, tackling ageism in healthcare may benefit not only each of us but society at large.

In this context, an international board of geriatrics experts convened an international working group to discuss ageism in healthcare.

## Ageism and Healthcare

In this document, we describe the effects of ageism in health and social care. We then propose actions that, through education and policies, can help to reduce ageism and promote healthy longevity.

For a long time, the medical approach to health focused on the diagnosis, management, and cure of single diseases. At a time when the proportion of older persons in the population was low and longevity was rare, middle-aged individuals with single, usually acute, diseases accounted for most of the patients seeking healthcare. The common, general paradigm was to treat each disease at the time of clinical emergence, prescribing therapy and sending patients home to heal or die. The mantra “one patient–one problem” has survived for hundreds of years. This approach ignored patients with multiple conditions, frailty and disability, considering these problems “normal consequences of aging,” “too complex,” and “unlikely to respond to care.” Until the last few decades, this approach was not yet amended by the evidence that prevention of diseases, health promotion, and productive employment matter into the oldest ages. With the aging of the population and a substantial reduction in mortality in older age, there has been a switch in the profile of patients accessing clinical services. These patients, indeed, are mostly affected by multiple chronic medical conditions that adversely affect their physical and cognitive function. To date, healthcare systems have only partially responded to the extensive transformation of population health, including the introduction of geriatrics as a medical specialty. The overarching mission of medical care remains rooted in the cure of a single disease, a strategy that conflicts with the already large and growing older population and the emergence of new patterns of morbidity and expanded health outcomes that require new models of care. Ageism is a substantial obstacle to both valuing and investing in the health and social care that matches the needs and opportunities for health of our aging population. Thus, it has become pressing to address the ageism that permeates medical care today, in order to adequately respond to the health needs and preferences of older persons.

Ageism is defined as “the stereotypes (how we think), prejudice (how we feel) and discrimination (how we act) directed towards others or oneself based on age.”

The demographic imperative of longevity and aging has led to an unprecedented expansion of the older population that is affected by chronic conditions and disabilities, making older people major healthcare users. This gradual transition now requires a profound and global transformation of the organization of healthcare for the individual as well as population-focused approaches to achieve healthy longevity. This will require the education of the healthcare and public health workforce and demands a stronger involvement of all providers who contribute to care, including social workers and informal caregivers. The literature supports a shift of healthcare systems toward integrated person-centered health and social care teams, who receive professional education on the appropriate care of older adults with varying combinations of conditions, life circumstances, and health priorities. Population health approaches need to incorporate goals for disease prevention and health promotion for older adults. Unfortunately, while the aging of the population is occurring globally, the specific needs of older persons are only recognized in a limited number of healthcare organizations and the university curricula of a few countries (institutional ageism).

The scope of this document is to point to actions that should be implemented now to minimize the adverse impact of ageism in healthcare and the unmet opportunities to prevent ill health in aging. Addressing ageism may require an initial investment but should eventually lead to substantial resource savings by avoiding unwanted illness or unhelpful care and aligning healthcare goals and care to the subjective preferences of each older adult. This transformation may involve the integration of less expensive and more generalizable rehabilitative, palliative, and social care rather than managing each medical condition separately.

We focus the discussion on a few urgent topics that we believe are critical and describe distinct manifestations of ageism in healthcare, public health, and their possible consequences. We also propose possible short-term and long-term solutions.

## Ageism in Healthcare: Major Manifestations, Consequences, and Actions

1. Endemic and internalized ageism—a barrier to adequate care
*Manifestation*: The universal undervaluing of older people permeates our culture and is at the root of ageism in healthcare. Ageism can also be internalized and eventually applied to oneself (self-directed ageism). In particular, older people may internalize the stereotype that older age is a period of inevitable disease and decline, a thought process that can impose barriers to engaging in health-promoting behaviors and accessing health and social care at an older age.
*Consequences*: All aspects of healthcare, from education to acute and long-term care, along with population-level prevention, remain outdated and inadequate to meet the expanding needs of the aging population, and ageism in healthcare is unlikely to be solved until endemic ageism is addressed. Older adults who internalize ageism experience worsening of physical and cognitive health and a shorter life expectancy than older adults with positive aging beliefs. Persons with negative aging stereotypes may disengage from healthy behaviors, such as taking prescribed medication, participating in physical activities, or following a healthy diet, because they will not see the potential gain from such behavior. Older people may also refuse to access health and social care services, because they believe that they do not deserve equal access and social care service or there is associated stigma.
*Action*: Education about aging and ageism in the general population, including in the current population of older people, is required to dismantle existing misconceptions and to promote healthy behaviors across the life course and to reinforce that every person has the same value regardless of age. Interventions that support positive aging beliefs are available that have been shown to improve aging perceptions and health, and these interventions should be broadly disseminated and supported.
2. Formative ageism—no education on older populations
*Manifestation*: Aging is ignored in curricula across educational programs for different health and social care providers. There is too little awareness that healthy aging is strongly influenced by the choices that we make over the whole life span.
*Consequences*: The lack of opportunities to learn about the aging process and older people in general can leave cultural norms unchallenged and result in negative attitudes against older patients. Most health and social care workers have not received educational opportunities around aging and older people and are therefore unprepared to respond to the preferences and healthcare and prevention needs of the older patients that they will eventually treat.
*Action*: Policies should be developed and implemented to ensure that aging becomes an integral part of any educational curriculum for health and social care professionals. Health and social care providers should also have the opportunity to participate in intergenerational activities involving older people, as this engagement has been demonstrated to effectively reduce ageism.
3. Clinical ageism—too much focus on treatment rather than prevention
*Manifestation*: Despite strong evidence that exposures and behaviors in early life can affect the aging process, as well as one’s health and function in later life, and that prevention and health promotion are effective into the oldest ages, aging is still widely considered a natural decay process that cannot be modified.
*Consequences*: Investment in healthcare is mostly directed to disease treatment, that is, care of diseases when they become clinically evident, rather than on prevention or health promotion over the life course. Progress in medical care has therefore mostly extended the length of life characterized by disease, with little effect on health expectancy and healthy longevity.
*Action*: Prioritizing preventive medicine and public health in earlier in life and over the life course will increase the probability of living a longer and healthier life for all. The main purpose of healthcare should be not only to cure diseases, but rather postpone the onset of diseases, frailty, and disability.
4. Clinical ageism: Focus on isolated treatment of individual diseases using evidence not applicable to older adults
*Manifestations*: Healthcare is primarily focused on diagnosing and managing individual diseases following guidelines based on evidence generated in younger adults with few conditions. Ageism might result in mis- or overtreatment, that is, provision of a treatment intervention that is based on disease-specific evidence generated in younger adults and extrapolated to older adults.
*Consequences*: This disease-based decision making results in interventions that may not be beneficial and may even be harmful and burdensome to older adults and does not address what matters most to them. For example, the exclusive focus on the treatment of individual diseases may lead to the adverse effects of polypharmacy, potentially harmful interventions, and unnecessary hospitalizations as individual diseases mount up and one’s overall function and preferences are ignored.
*Action*: In addition to acquiring and using therapeutic evidence on functional, symptom-based, and quality of life outcomes in older adults with multiple conditions, care should focus on identifying the specific health outcome goals of older adults and implementing realistic care aligned with meeting these goals. Treatment should be decided in collaboration with the patient and in the context of their comorbidity, functional capacity, social support, and living environment.
5. Clinical ageism—Lack of involvement in care choices
*Manifestation*: Clinical decision making does not adequately consider alternatives of care that may better align with subjective priorities and preferences of older patients, including the decision to withhold treatment to avoid iatrogenic harm. For example, maximizing function and preventing frailty, and disability are often appropriate primary targets of interventions, but older people may not be involved in the development of a care plan with these goals in mind.
*Consequences*: Treatment choices are offered to older people and their caregivers without informing them of other possible choices goals and choices that may better match subjective priorities and preferences. Poor adherence might occur as a consequence of the limited involvement of older persons in treatment decision making.
*Action*: Identifying the health outcome goals of each individual using a person-centered care approach and providing a comprehensive explanation of the consequence(s) of alternative therapeutic choices to allow shared decision making should become an integral component of medical education and practice. This is particularly important for older patients who are often affected by complex health problems not amenable to a “cure.” Quaternary prevention (“primum non nocere”), including deprescribing when appropriate, and patient-reported outcomes and experiences (person-reported outcome measure and patient-reported experience measures) should receive proper attention in medical and paramedical education. Caregivers should be involved in clinical decision making, as appropriate, taking into consideration the preferences and priorities of those they care for.
6. Clinical ageism—denying available treatment or preventative measures
*Manifestation*: Ageism leads to an age-based, unjustified and discriminatory exclusion of older patients from treatment that can be life-saving or essential to preserve function and/or quality of life. Older people also experience discrimination in their access to preventive measures, such as mammography screening.
*Consequences*: Older patients, based on their chronological age, are less likely to be eligible to receive intensive care or complex medical and surgical treatment, regardless of the severity of their baseline condition, or their level of intrinsic capacity.
*Action*: Considerations about biological age and function and individual health goals and care preferences, instead of chronological age, should guide treatment goals and choices (see also point 4 above) and allocation of treatment resources to the geriatric population. Legislation should be devised and appropriately implemented to ensure that healthcare rationing by age is prohibited. High-quality and dignified end-of-life care should be guaranteed when appropriate.
7. Ageism in research—lack of evidence-based medicine
*Manifestation*: Older patients in general and particularly those with multiple physical and mental conditions and disabilities are often excluded from clinical trials that test the effectiveness and safety of pharmacological and nonpharmacological interventions. This is even true for trials testing interventions for conditions that are more prevalent in older age. When data *are* collected on older people, they are usually not highlighted but instead are buried in a single age group of over 60 or 65 years of age, therefore hiding the enormous diversity among older individuals. This undermines the validity of the results for most older adults and also leads to broad underinvestment in bio-gerontological aging research.
*Consequence*: Validation of the efficacy and safety of treatments often does not apply to older patients, especially those with clinical and social complexity. Additionally, tools that can be used on a large scale for risk stratification are lacking, preventing older people from the possibility of receiving proper prognostic assessment and getting access to specific care and clinical pathways.
*Action*: Older patients should be included in clinical trials aimed to test interventions that may become beneficial to them. Policies could be generated to promote and ensure adequate representation of older people in research. Data should be more extensively stratified by age and health status measures and require functional, symptom, and quality of life outcomes in addition to disease-specific outcomes and survival. More research is needed to develop new study designs (e.g., pragmatic trials) and outcomes that enable a more inclusive participation regardless of age and comorbid conditions.
8. Healthcare system ageism—disconnection between healthcare settings and the community
*Manifestation*: There is a lack of communication and connection between the different settings and the health and social care professionals that provide care for the same person, especially for older persons with cognitive impairment who cannot advocate for themselves.
*Consequences*: A lack of integration and continuity between medical and social care, including informal care, increases the risk of inadequate medical management of older patients with multiple chronic diseases, cognitive impairment, polypharmacy, frailty, and/or disability. Such a lack of integration and communication may result in adverse health outcomes, inappropriate polypharmacy and drug interactions, redundancy of diagnostic and therapeutic interventions, and multiple readmissions to different care facilities.
*Action*: There is a need for integrated and coordinated health and social care networks to promote more comprehensive and effective assistance. Geriatric medicine may play a pivotal role in the oversight of this process, favoring connections and integration between specialized settings (e.g., by designing, overseeing, and coordinating a care plan from acute care, to subintensive care, to rehabilitation, and then to long-term care solutions) and primary care services.
9. Clinical ageism—deprioritized in acute and emergency care delivery
*Manifestations*: It is well known that acute medical problems in older patients often trigger a rapid deterioration of health and function, often leading to a decision for hospital admission from the emergency room. However, older people with acute problems are not given priority for triage and treatment. For example, an ageist paradox is that older patients are often left waiting for many more hours in emergency rooms than younger patients.
*Consequences*: Acute problems that could have been successfully and rapidly treated become catastrophic and irreversible medical conditions that substantially change the health and functional trajectories of the patient (e.g., untreated urinary retention can evolve into delirium with nursing home admission and subsequently precipitate dementia).
*Action*: An expanded role of primary and community care, a better integration of services, the establishment of a surveillance system for the frailest persons, and the creation of a procedure for rapid activation of social and caregiver support can minimize the use of emergency rooms. When admission to the emergency room cannot be avoided, older patients should receive priority attention and age-appropriate care to avoid irreversible decline of health conditions. Frail older patients should be treated rapidly, independently of evident clinical instability, because of their high risk of deterioration during emergency department stay.
10. Ageism in the design and operation of healthcare facilities
*Manifestations*: Healthcare facilities often do not include spaces specifically tailored to the needs of older patients, such as those that facilitate early rehabilitation, orientation and socialization. Furthermore, hospitals are designed to keep patients relatively immobile and isolated in bed, rather than allow them to engage in toileting, physical activity, socialization, and uninterrupted sleep that can promote recovery and prevent complications such as delirium, falls, deconditioning, incontinence, and depression.
*Consequences*: The inadequate environments of healthcare facilities lead to a high incidence of complications (e.g., delirium, immobilization syndrome) and acute loss of physical and cognitive function that could have been avoided if they provided more home-like spaces and activities.
*Action*: Every healthcare facility should include age-friendly environments that optimize the care of older patients, for example, dedicated areas with quiet rooms, accessible bathrooms, indirect lighting, warmth, and spaces promoting early rehabilitation, occupational therapy, and family visits.
11. Ageism in healthcare access
*Manifestation*: Ageism is linked to reduced healthcare access. Older patients often have limited access to healthcare, they may lack suitable transportation, and they may not be able to afford an alternative form of care.
*Consequences*: Older adults may be more likely to face catastrophic medical costs, as they may experience multiple and simultaneous health and social issues and may need to travel long distances to access care. This is particularly the case in countries with no universal health coverage and where care is mostly provided at secondary and tertiary levels. These factors differently affect the health, quality of life, and survival in older compared to younger persons, regardless of their background health status.
*Action*: Access to healthcare should be guaranteed to older people and especially those with disability, frailty, social isolation, and poor socioeconomic status. Care should encompass oral health, eye health, hearing devices, and other services usually provided outside the public healthcare system. Public transportation to healthcare facilities should be accessible and affordable for older persons.
12. Ageism in healthcare technologies
*Manifestation*: Ageism in healthcare technologies lies in misconceptions about older individuals’ abilities to understand and use digital technologies, which are typically designed for younger adults. Of note, the use of digital technology is becoming a main path of communication between individuals and the healthcare system. At the same time, there is a risk that artificial intelligence (AI) technologies used in medicine and public health exacerbate or introduce new forms of ageism if left unchecked. AI-based clinical prediction and decision-making tools will be based on research data from clinical trials in which older adults, particularly those with multimorbidity or functional limitations, were largely excluded, or on clinical data that lack the functional outcomes of particular importance to older adults and ignore the individual health priorities of older adults.
*Consequences*: The design of technological devices and software often fails to consider the specific needs of older adults, typically being manufactured in small sizes and with complex interfaces that are not user friendly for people with arthritis or visual impairment. Use of existing technologies may also be negatively affected by cost, inadequate training, poor social support, or limited internet connectivity. Older adults are thus denied the benefits of healthcare technological devices and online platforms, leading to unequal access to technology-based monitoring and care. The inappropriate application of AI-generated prediction and clinical decision-making tools will likely exacerbate the inappropriate care of older adults described in points 4, 5, and 7.
*Action*: It is crucial to raise awareness among healthcare providers, technology developers, and policymakers about older adults’ specific needs related to technology. Engagement of older adults in the design and implementation of healthcare technologies, including AI, may help to develop age-friendly tools that allow older people to benefit from technology-based care strategies and engage in health promotion resources. Data from older adults across the full spectrum of health and functional conditions must be included in the data used to generate clinical prediction and decision-making models if results are to be applied to older adults. Functional status and individual health priorities need to become standard data elements in electronic medical records.

## Final Considerations

Ageism is pervasive and involves most aspects of our life. We learn early that being “young” as opposite to being “old” is a positive value and this ageist cultural view persists over the whole lifespan. The “fear” of becoming old and the “surprise” of reaching old age are only a few examples of the “ageist” imprinting that we carry with us. Such negative cultural stereotypes of aging have severe consequence on the lives of older persons, who tend to be marginalized and left out of many opportunities just because of “old age.” Mandatory retirement is imposed on people who may not have any physical or cognitive impairment and can still make major contributions to the community or at work. Perhaps the most burning consequence of cultural ageism is the lack recognition of the value and special needs of older people by healthcare systems, and a failure to make the necessary changes despite the demographic transformation that is occurring in every country in the world. In this document, we identified the main instances where ageism permeates health and social care, resulting in suboptimal care for this rising portion of the population. We purposely focused on a few specific topics, fully aware that this document is not a comprehensive inventory of the many ways by which ageism hampers the health and care of older persons and reduces their ability to maximize their quality of life and contribution to society. Instead, we focused on what we see as the main manifestations of ageism in healthcare that, if addressed, may be transformative on the quality of care provided to older persons, and their quality of life. It is the view of the working group that this is a living document that will evolve over time as our understanding of the manifestations and effect of ageism in healthcare improves, through our own experiences, the reading of expanding literature and, hopefully, the many comments and suggestions that will come from those that critically read our recommendations.

